# 3-[3-Methyl-4-(4-nitro­benzyl­idene­amino)-5-sulfanyl­idene-4,5-dihydro-1*H*-1,2,4-triazol-1-yl]-1,3-diphenyl­propan-1-one dichloro­methane monosolvate

**DOI:** 10.1107/S1600536811043777

**Published:** 2011-10-29

**Authors:** Wei Wang, Wei-Min Jia, Chao Xu, Wen-Peng Wu, Qing-Lei Liu

**Affiliations:** a School of Perfume and Aroma Technology, Shanghai Institute of Technology, Shanghai 200235, People’s Republic of China; b School of Chemical Engineering, University of Science and Technology Liaoning, Anshan 114051, People’s Republic of China

## Abstract

In the title compound, C_25_H_21_N_5_O_3_S·CH_2_Cl_2_, the dichloro­methane solvent mol­ecule is disordered over four positions, with an occupancy ratio of 0.271 (3):0.3884 (18):0.298 (2):0.0424 (15). The 1,2,4-triazole ring makes dihedral angles of 47.3 (2)/87.3 (2) and 3.6 (2)° with the phenyl and nitro­phenyl rings, respectively. An intra­molecular C—H⋯S hydrogen bond results in the formation of an almost planar six-membered ring [r.m.s. derivation = 0.0051 (2) Å]. Inter­molecular C—H⋯O hydrogen bonding consolidates the structure.

## Related literature

For crystal structures related to 1,2,4-triazole-5(4*H*)-thione, see: Al-Tamimi *et al.* (2010 ▶); Fun *et al.* (2009 ▶); Gao *et al.* (2011 ▶); Tan *et al.* (2010 ▶); Wang *et al.* (2011 ▶); Zhao *et al.* (2010 ▶).
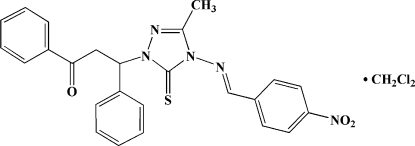


## Experimental

### 

#### Crystal data

C_25_H_21_N_5_O_3_S·CH_2_Cl_2_
*M* *_r_* = 556.45
Triclinic, 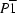

*a* = 8.9880 (13) Å
*b* = 11.4440 (15) Å
*c* = 14.8604 (18) Å
α = 70.212 (11)°
β = 88.973 (13)°
γ = 67.020 (9)°
*V* = 1312.6 (3) Å^3^
*Z* = 2
Mo *K*α radiation
μ = 0.37 mm^−1^
*T* = 113 K
0.26 × 0.24 × 0.20 mm

#### Data collection

Rigaku Saturn CCD diffractometer
Absorption correction: multi-scan (*CrystalClear*; Rigaku/MSC, 2005 ▶) *T*_min_ = 0.911, *T*_max_ = 0.931
16932 measured reflections
6203 independent reflections
4100 reflections with *I* > 2σ(*I*)
*R*_int_ = 0.035

#### Refinement

*R*[*F* ^2^ > 2σ(*F* ^2^)] = 0.052
*wR*(*F* ^2^) = 0.159
*S* = 1.07
6203 reflections
396 parameters
91 restraints
H-atom parameters constrained
Δρ_max_ = 0.27 e Å^−3^
Δρ_min_ = −0.41 e Å^−3^

### 

Data collection: *CrystalClear* (Rigaku/MSC, 2005 ▶); cell refinement: *CrystalClear*; data reduction: *CrystalClear*; program(s) used to solve structure: *SHELXS97* (Sheldrick, 2008 ▶); program(s) used to refine structure: *SHELXL97* (Sheldrick, 2008 ▶); molecular graphics: *SHELXTL* (Sheldrick, 2008 ▶); software used to prepare material for publication: *CrystalStructure* (Rigaku/MSC, 2007 ▶).

## Supplementary Material

Crystal structure: contains datablock(s) global, I. DOI: 10.1107/S1600536811043777/zk2024sup1.cif

Structure factors: contains datablock(s) I. DOI: 10.1107/S1600536811043777/zk2024Isup2.hkl

Supplementary material file. DOI: 10.1107/S1600536811043777/zk2024Isup3.cml

Additional supplementary materials:  crystallographic information; 3D view; checkCIF report

## Figures and Tables

**Table 1:** Hydrogen-bond geometry (Å, °)

*D*—H⋯*A*	*D*—H	H⋯*A*	*D*⋯*A*	*D*—H⋯*A*
C19—H19⋯S1	0.95	2.43	3.199 (3)	137
C18—H18*B*⋯O1^i^	0.98	2.55	3.443 (4)	152
C22—H22⋯O1^ii^	0.95	2.34	3.202 (3)	151

Symmetry codes: (i) 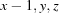; (ii) 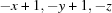.

**Fig. 1.**
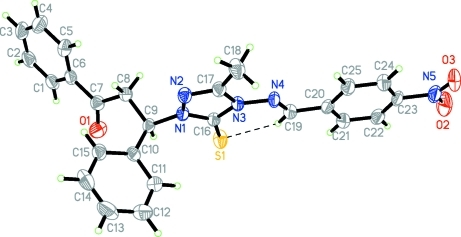
View of the molecule of (I) showing the atom-labelling scheme. Displacement ellipsoids are drawn at the 60% probability level. The disordered dichloromethane molecule is omitted.

**Fig. 2.**
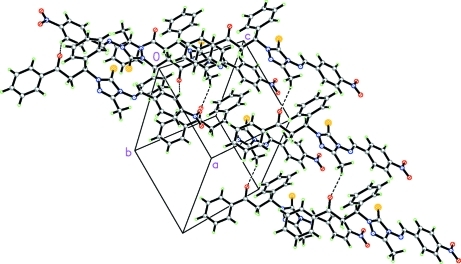
Packing diagram of the crystal structure. The disordered dichloromethane molecule is omitted.

## supplementary crystallographic information

### Comment

In continuation of our structural studies of derivatives of Mannich bases synthesized from amino heterocycles and aromatic aldehydes in our group (Wang *et al.*, 2011), we present here the crystal structure of the title compound named 3-[4-(4-Nitrobenzylideneamino)-5-thioxo-3-(4-tolyl)-4,5- dihydro-1*H*-1,2,4-triazol-1-yl]-1,3-diphenylpropan-1-one.

The bond lengths and angles in title compound are found to have normal values comparable with those reported in related 1,2,4-triazole- 5(4*H*)-thione derivatives (Al-Tamimi *et al.*, 2010; Fun *et al.*, 2009; Tan *et al.*, 2010; Wang *et al.*, 2011). An intramolecular C—H···S hydrogen bond results in the formation of a planar [an r.m.s. deviation of 0.0051 (2) Å] six-membered ring (Table 1) and the maxmium deviation of 0.0088 (2) Å for atom N4. The 1,2,4-triazole ring is almost planar with an r.m.s. deviation of 0.0039 (2) Å and the maxmium deviation of 0.0061 (2) Å for atom N1. The 1,2,4-triazole ring mean plane forms the dihedral angles of 47.3 (2), 87.3 (2) and 3.6 (2)° with two phenyl rings (C1–C6 and C10–C15) and nitrophenyl ring, respectively. Two C atoms in the 1,2,4-triazole ring show distorted C*sp*^2^ hybridization states with the bond angles of 101.95 (16)° (N1—C16—N3), 130.44 (15)° (N3—C16—S1), 110.44 (18)° (N2—C17—N3) and 25.88 (19)° (N3—C17—C18), which are similar to those of similar reported triazole derivatives (Zhao *et al.*, 2010; Gao *et al.*, 2011).

In the crystal structure, weak intermolecular C—H···O hydrogen bonds (Table 1) are observed and consolidate the crystal structure.

### Experimental

The title compound was synthesized with the reaction of 4-nitrobenzaldehyde (2.0 mmol) and 3-(4-amino-3-methyl-5-thioxo-4,5-dihydro-1*H*-1,2,4-triazol-1-yl)-1,3-diphenylpropan-1-one (2.0 mmol) by refluxing in ethanol. The reaction progress was monitored *via* TLC. The resulting precipitate was filtered off, washed with cold ethanol, dried and purified to give the target product as a colorless solid in 74% yield. Crystals of title compound suitable for single-crystal X-ray analysis were grown by slow evaporation of a solution in dichloromethanle–ethanol (1:1).

### Refinement

All H atoms were positioned geometrically and refined as riding (C—H = 0.95–1.00 Å) on their parent atoms, with *U*_iso_(H) = 1.2*U*_eq_(C) or 1.5*U*_eq_(C). One molecule of solvent dichloromethane is present in the asymmetric unit. This was refined as disordered over four positions with occupancies of 0.271 (3):0.3884 (18):0.298 (2):0.0424 (15).

### Crystal data

**Table:**

C_25_H_21_N_5_O_3_S·CH_2_Cl_2_	*Z* = 2
*M**_r_* = 556.45	*F*(000) = 576
Triclinic, *P*1	*D*_x_ = 1.408 Mg m^−^^3^
Hall symbol: -P 1	Mo *K*α radiation, λ = 0.71073 Å
*a* = 8.9880 (13) Å	Cell parameters from 4632 reflections
*b* = 11.4440 (15) Å	θ = 2.1–27.9°
*c* = 14.8604 (18) Å	µ = 0.37 mm^−^^1^
α = 70.212 (11)°	*T* = 113 K
β = 88.973 (13)°	Prism, colourless
γ = 67.020 (9)°	0.26 × 0.24 × 0.20 mm
*V* = 1312.6 (3) Å^3^	

### Data collection

**Table:**

Rigaku Saturn CCD diffractometer	6203 independent reflections
Radiation source: rotating anode	4100 reflections with *I* > 2σ(*I*)
multilayer	*R*_int_ = 0.035
Detector resolution: 14.22 pixels mm^-1^	θ_max_ = 27.9°, θ_min_ = 2.1°
φ and ω scans	*h* = −11→11
Absorption correction: multi-scan (*CrystalClear*; Rigaku/MSC, 2005)	*k* = −15→15
*T*_min_ = 0.911, *T*_max_ = 0.931	*l* = −19→18
16932 measured reflections	

### Refinement

**Table:**

Refinement on *F*^2^	Primary atom site location: structure-invariant direct methods
Least-squares matrix: full	Secondary atom site location: difference Fourier map
*R*[*F*^2^ > 2σ(*F*^2^)] = 0.052	Hydrogen site location: inferred from neighbouring sites
*wR*(*F*^2^) = 0.159	H-atom parameters constrained
*S* = 1.07	*w* = 1/[σ^2^(*F*_o_^2^) + (0.0904*P*)^2^] where *P* = (*F*_o_^2^ + 2*F*_c_^2^)/3
6203 reflections	(Δ/σ)_max_ = 0.003
396 parameters	Δρ_max_ = 0.27 e Å^−^^3^
91 restraints	Δρ_min_ = −0.41 e Å^−^^3^

### Special details

**Table:**

Geometry. All e.s.d.'s (except the e.s.d. in the dihedral angle between two l.s. planes) are estimated using the full covariance matrix. The cell e.s.d.'s are taken into account individually in the estimation of e.s.d.'s in distances, angles and torsion angles; correlations between e.s.d.'s in cell parameters are only used when they are defined by crystal symmetry. An approximate (isotropic) treatment of cell e.s.d.'s is used for estimating e.s.d.'s involving l.s. planes.
Refinement. Refinement of *F*^2^ against ALL reflections. The weighted *R*-factor *wR* and goodness of fit *S* are based on *F*^2^, conventional *R*-factors *R* are based on *F*, with *F* set to zero for negative *F*^2^. The threshold expression of *F*^2^ > σ(*F*^2^) is used only for calculating *R*-factors(gt) *etc*. and is not relevant to the choice of reflections for refinement. *R*-factors based on *F*^2^ are statistically about twice as large as those based on *F*, and *R*-factors based on ALL data will be even larger.

### Fractional atomic coordinates and isotropic or equivalent isotropic displacement parameters (Å^2^)

**Table:**

	*x*	*y*	*z*	*U*_iso_*/*U*_eq_	Occ. (<1)
S1	0.48389 (7)	0.55051 (6)	0.09792 (4)	0.03327 (18)	
O1	0.91577 (19)	0.35679 (16)	0.36980 (12)	0.0382 (4)	
O2	−0.3675 (2)	0.7421 (2)	−0.32320 (14)	0.0570 (5)	
O3	−0.5429 (2)	0.7897 (2)	−0.22653 (14)	0.0565 (5)	
N1	0.4215 (2)	0.54864 (17)	0.27759 (11)	0.0220 (4)	
N2	0.2982 (2)	0.57257 (18)	0.33400 (12)	0.0252 (4)	
N4	0.0767 (2)	0.63610 (17)	0.12001 (12)	0.0253 (4)	
N5	−0.4037 (3)	0.75374 (19)	−0.24612 (15)	0.0387 (5)	
C1	1.0752 (3)	0.1110 (2)	0.52278 (17)	0.0316 (5)	
H1	1.1363	0.1371	0.4730	0.038*	
C2	1.1508 (3)	−0.0061 (2)	0.60281 (18)	0.0376 (6)	
H2	1.2634	−0.0614	0.6072	0.045*	
C3	1.0636 (3)	−0.0431 (2)	0.67630 (18)	0.0390 (6)	
H3	1.1169	−0.1228	0.7316	0.047*	
C4	0.8977 (3)	0.0356 (2)	0.67000 (17)	0.0401 (6)	
H4	0.8377	0.0097	0.7206	0.048*	
C5	0.8204 (3)	0.1531 (2)	0.58855 (16)	0.0320 (5)	
H5	0.7072	0.2070	0.5835	0.038*	
C6	0.9085 (2)	0.1913 (2)	0.51510 (15)	0.0262 (4)	
C7	0.8314 (3)	0.3141 (2)	0.42531 (15)	0.0270 (5)	
C8	0.6478 (2)	0.3800 (2)	0.40255 (15)	0.0270 (5)	
H8A	0.5998	0.3976	0.4596	0.032*	
H8B	0.6098	0.3167	0.3888	0.032*	
C9	0.5887 (2)	0.5135 (2)	0.31618 (14)	0.0232 (4)	
H9	0.6596	0.4983	0.2650	0.028*	
C10	0.5934 (2)	0.6341 (2)	0.33558 (15)	0.0246 (4)	
C11	0.5370 (3)	0.7600 (2)	0.25927 (17)	0.0331 (5)	
H11	0.5027	0.7664	0.1970	0.040*	
C12	0.5308 (3)	0.8754 (3)	0.2736 (2)	0.0440 (6)	
H12	0.4908	0.9606	0.2217	0.053*	
C13	0.5833 (3)	0.8658 (3)	0.3641 (2)	0.0469 (7)	
H13	0.5785	0.9446	0.3743	0.056*	
C14	0.6420 (3)	0.7426 (3)	0.43870 (19)	0.0406 (6)	
H14	0.6787	0.7362	0.5004	0.049*	
C15	0.6480 (3)	0.6268 (2)	0.42456 (16)	0.0284 (5)	
H15	0.6902	0.5417	0.4766	0.034*	
C16	0.3708 (2)	0.56787 (19)	0.18648 (14)	0.0228 (4)	
N3	0.2032 (2)	0.60395 (16)	0.18804 (12)	0.0223 (4)	
C17	0.1673 (2)	0.6053 (2)	0.27919 (14)	0.0249 (4)	
C18	0.0009 (3)	0.6404 (3)	0.30726 (17)	0.0363 (5)	
H18A	0.0046	0.6354	0.3744	0.054*	
H18B	−0.0412	0.5762	0.3008	0.054*	
H18C	−0.0707	0.7329	0.2652	0.054*	
C19	0.1070 (3)	0.6337 (2)	0.03578 (15)	0.0275 (5)	
H19	0.2148	0.6118	0.0195	0.033*	
C20	−0.0282 (3)	0.6655 (2)	−0.03495 (15)	0.0264 (5)	
C21	0.0061 (3)	0.6678 (2)	−0.12770 (16)	0.0303 (5)	
H21	0.1140	0.6493	−0.1430	0.036*	
C22	−0.1174 (3)	0.6972 (2)	−0.19751 (16)	0.0318 (5)	
H22	−0.0955	0.6998	−0.2608	0.038*	
C23	−0.2716 (3)	0.7224 (2)	−0.17278 (15)	0.0297 (5)	
C24	−0.3097 (3)	0.7184 (2)	−0.08119 (16)	0.0324 (5)	
H24	−0.4176	0.7352	−0.0662	0.039*	
C25	−0.1863 (3)	0.6893 (2)	−0.01263 (16)	0.0311 (5)	
H25	−0.2093	0.6855	0.0507	0.037*	
Cl1	0.8120 (5)	1.0389 (4)	−0.0808 (3)	0.0362 (8)	0.271 (3)
Cl2	0.7811 (5)	1.0728 (4)	0.0934 (3)	0.0640 (12)	0.271 (3)
C26	0.6696 (14)	1.0852 (19)	−0.0030 (9)	0.094 (3)	0.271 (3)
H26A	0.5868	1.1794	−0.0348	0.112*	0.271 (3)
H26B	0.6137	1.0229	0.0170	0.112*	0.271 (3)
C27	0.7212 (11)	0.9953 (13)	0.0241 (7)	0.094 (3)	0.3884 (18)
H27A	0.7977	0.9021	0.0305	0.112*	0.3884 (18)
H27B	0.7413	1.0585	−0.0343	0.112*	0.3884 (18)
Cl3	0.5337 (2)	1.0078 (2)	0.00310 (16)	0.0435 (6)	0.3884 (18)
Cl4	0.7722 (3)	1.0250 (3)	0.11800 (18)	0.0621 (7)	0.3884 (18)
C28	0.9267 (13)	0.9766 (13)	0.0362 (7)	0.079 (4)	0.298 (2)
H28A	0.9329	0.8830	0.0550	0.095*	0.298 (2)
H28B	0.8410	1.0225	0.0705	0.095*	0.298 (2)
Cl6	1.1012 (7)	0.9588 (6)	0.0885 (4)	0.0887 (19)	0.298 (2)
Cl5	0.8513 (7)	1.0491 (5)	−0.0756 (4)	0.0586 (13)	0.298 (2)
Cl7	1.0305 (17)	0.9999 (14)	−0.0990 (9)	0.0362 (8)	0.0424 (15)
Cl8	1.176 (3)	1.000 (2)	0.0685 (12)	0.0362 (8)	0.0424 (15)
C29	1.185 (7)	0.908 (4)	−0.004 (4)	0.079 (4)	0.0424 (15)
H29A	1.2915	0.8843	−0.0290	0.095*	0.0424 (15)
H29B	1.1758	0.8219	0.0345	0.095*	0.0424 (15)

### Atomic displacement parameters (Å^2^)

**Table:**

	*U*^11^	*U*^22^	*U*^33^	*U*^12^	*U*^13^	*U*^23^
S1	0.0257 (3)	0.0509 (4)	0.0245 (3)	−0.0139 (3)	0.0057 (2)	−0.0174 (3)
O1	0.0242 (8)	0.0376 (9)	0.0401 (9)	−0.0080 (7)	0.0043 (7)	−0.0043 (8)
O2	0.0592 (13)	0.0715 (13)	0.0390 (11)	−0.0221 (11)	−0.0150 (9)	−0.0229 (10)
O3	0.0368 (11)	0.0656 (13)	0.0511 (12)	−0.0185 (10)	−0.0200 (9)	−0.0036 (10)
N1	0.0193 (8)	0.0270 (9)	0.0185 (8)	−0.0089 (7)	0.0019 (6)	−0.0073 (7)
N2	0.0218 (8)	0.0323 (9)	0.0213 (8)	−0.0107 (7)	0.0031 (7)	−0.0098 (8)
N4	0.0229 (9)	0.0259 (9)	0.0234 (9)	−0.0079 (7)	−0.0056 (7)	−0.0065 (7)
N5	0.0428 (13)	0.0293 (10)	0.0365 (12)	−0.0141 (9)	−0.0164 (10)	−0.0029 (9)
C1	0.0258 (11)	0.0290 (11)	0.0397 (13)	−0.0120 (9)	−0.0047 (10)	−0.0106 (10)
C2	0.0275 (12)	0.0291 (12)	0.0502 (15)	−0.0093 (10)	−0.0122 (11)	−0.0088 (11)
C3	0.0400 (14)	0.0278 (12)	0.0373 (13)	−0.0088 (11)	−0.0169 (11)	−0.0024 (10)
C4	0.0469 (15)	0.0356 (13)	0.0289 (12)	−0.0140 (12)	−0.0045 (11)	−0.0039 (11)
C5	0.0299 (12)	0.0309 (11)	0.0284 (11)	−0.0067 (10)	−0.0033 (9)	−0.0090 (10)
C6	0.0241 (10)	0.0240 (10)	0.0291 (11)	−0.0086 (9)	−0.0046 (9)	−0.0092 (9)
C7	0.0244 (10)	0.0265 (10)	0.0292 (11)	−0.0091 (9)	0.0017 (9)	−0.0102 (9)
C8	0.0232 (10)	0.0259 (10)	0.0277 (11)	−0.0104 (9)	−0.0010 (8)	−0.0043 (9)
C9	0.0170 (9)	0.0270 (10)	0.0213 (10)	−0.0075 (8)	−0.0002 (8)	−0.0050 (8)
C10	0.0185 (9)	0.0289 (11)	0.0269 (10)	−0.0110 (8)	0.0031 (8)	−0.0090 (9)
C11	0.0350 (12)	0.0322 (12)	0.0311 (12)	−0.0174 (10)	−0.0005 (10)	−0.0058 (10)
C12	0.0467 (15)	0.0329 (13)	0.0494 (16)	−0.0211 (12)	0.0000 (12)	−0.0055 (12)
C13	0.0467 (15)	0.0402 (14)	0.0666 (19)	−0.0257 (12)	0.0034 (14)	−0.0248 (14)
C14	0.0380 (13)	0.0565 (16)	0.0431 (14)	−0.0270 (12)	0.0052 (11)	−0.0275 (13)
C15	0.0243 (10)	0.0351 (12)	0.0259 (11)	−0.0135 (9)	0.0025 (9)	−0.0095 (9)
C16	0.0223 (10)	0.0215 (10)	0.0231 (10)	−0.0084 (8)	−0.0002 (8)	−0.0068 (8)
N3	0.0202 (8)	0.0246 (9)	0.0206 (8)	−0.0082 (7)	0.0001 (7)	−0.0073 (7)
C17	0.0244 (10)	0.0293 (11)	0.0211 (10)	−0.0116 (9)	0.0029 (8)	−0.0083 (9)
C18	0.0237 (11)	0.0567 (15)	0.0323 (12)	−0.0167 (11)	0.0073 (9)	−0.0204 (12)
C19	0.0260 (11)	0.0267 (11)	0.0271 (11)	−0.0102 (9)	−0.0011 (9)	−0.0068 (9)
C20	0.0283 (11)	0.0227 (10)	0.0242 (10)	−0.0095 (9)	−0.0038 (9)	−0.0043 (9)
C21	0.0310 (11)	0.0334 (11)	0.0263 (11)	−0.0145 (10)	0.0004 (9)	−0.0088 (9)
C22	0.0378 (13)	0.0306 (11)	0.0243 (11)	−0.0139 (10)	−0.0026 (9)	−0.0063 (9)
C23	0.0349 (12)	0.0217 (10)	0.0256 (11)	−0.0105 (9)	−0.0122 (9)	−0.0007 (9)
C24	0.0265 (11)	0.0318 (12)	0.0346 (12)	−0.0101 (10)	−0.0023 (9)	−0.0088 (10)
C25	0.0299 (11)	0.0339 (12)	0.0254 (11)	−0.0113 (10)	−0.0012 (9)	−0.0078 (10)
Cl1	0.0368 (10)	0.0373 (10)	0.0345 (9)	−0.0143 (6)	0.0046 (6)	−0.0140 (6)
Cl2	0.0677 (15)	0.0600 (14)	0.0678 (15)	−0.0239 (10)	0.0039 (9)	−0.0294 (10)
C26	0.094 (3)	0.093 (3)	0.093 (3)	−0.0377 (14)	0.0128 (9)	−0.0339 (13)
C27	0.094 (3)	0.093 (3)	0.093 (3)	−0.0377 (14)	0.0128 (9)	−0.0339 (13)
Cl3	0.0459 (10)	0.0446 (8)	0.0421 (7)	−0.0168 (7)	0.0075 (8)	−0.0201 (6)
Cl4	0.0617 (10)	0.0766 (11)	0.0486 (10)	−0.0279 (8)	0.0057 (7)	−0.0234 (8)
C28	0.079 (4)	0.079 (4)	0.079 (4)	−0.0324 (16)	0.0104 (9)	−0.0286 (15)
Cl6	0.089 (2)	0.088 (2)	0.090 (2)	−0.0372 (12)	0.0078 (10)	−0.0314 (11)
Cl5	0.0574 (16)	0.0577 (15)	0.0607 (15)	−0.0229 (10)	0.0094 (10)	−0.0221 (10)
Cl7	0.0368 (10)	0.0373 (10)	0.0345 (9)	−0.0143 (6)	0.0046 (6)	−0.0140 (6)
Cl8	0.0368 (10)	0.0373 (10)	0.0345 (9)	−0.0143 (6)	0.0046 (6)	−0.0140 (6)
C29	0.079 (4)	0.079 (4)	0.079 (4)	−0.0324 (16)	0.0104 (9)	−0.0286 (15)

### Geometric parameters (Å, °)

**Table:**

S1—C16	1.666 (2)	C14—C15	1.391 (3)
O1—C7	1.220 (3)	C14—H14	0.9500
O2—N5	1.221 (3)	C15—H15	0.9500
O3—N5	1.219 (3)	C16—N3	1.399 (2)
N1—C16	1.353 (2)	N3—C17	1.391 (2)
N1—N2	1.375 (2)	C17—C18	1.481 (3)
N1—C9	1.467 (2)	C18—H18A	0.9800
N2—C17	1.293 (3)	C18—H18B	0.9800
N4—C19	1.283 (3)	C18—H18C	0.9800
N4—N3	1.383 (2)	C19—C20	1.469 (3)
N5—C23	1.475 (3)	C19—H19	0.9500
C1—C2	1.383 (3)	C20—C25	1.394 (3)
C1—C6	1.401 (3)	C20—C21	1.401 (3)
C1—H1	0.9500	C21—C22	1.391 (3)
C2—C3	1.379 (4)	C21—H21	0.9500
C2—H2	0.9500	C22—C23	1.371 (3)
C3—C4	1.394 (4)	C22—H22	0.9500
C3—H3	0.9500	C23—C24	1.388 (3)
C4—C5	1.398 (3)	C24—C25	1.379 (3)
C4—H4	0.9500	C24—H24	0.9500
C5—C6	1.388 (3)	C25—H25	0.9500
C5—H5	0.9500	Cl1—C26	1.757 (9)
C6—C7	1.496 (3)	Cl2—C26	1.696 (9)
C7—C8	1.510 (3)	C26—H26A	0.9900
C8—C9	1.525 (3)	C26—H26B	0.9900
C8—H8A	0.9900	C27—Cl4	1.656 (8)
C8—H8B	0.9900	C27—Cl3	1.661 (8)
C9—C10	1.518 (3)	C27—H27A	0.9900
C9—H9	1.0000	C27—H27B	0.9900
C10—C15	1.383 (3)	C28—Cl5	1.606 (8)
C10—C11	1.401 (3)	C28—Cl6	1.671 (8)
C11—C12	1.388 (3)	C28—H28A	0.9900
C11—H11	0.9500	C28—H28B	0.9900
C12—C13	1.386 (4)	Cl7—C29	1.718 (11)
C12—H12	0.9500	Cl8—C29	1.723 (11)
C13—C14	1.370 (4)	C29—H29A	0.9900
C13—H13	0.9500	C29—H29B	0.9900
			
C16—N1—N2	113.97 (16)	N1—C16—S1	127.61 (15)
C16—N1—C9	125.89 (17)	N3—C16—S1	130.44 (15)
N2—N1—C9	120.04 (16)	N4—N3—C17	118.17 (16)
C17—N2—N1	105.18 (16)	N4—N3—C16	133.38 (17)
C19—N4—N3	119.39 (18)	C17—N3—C16	108.45 (16)
O3—N5—O2	123.6 (2)	N2—C17—N3	110.44 (18)
O3—N5—C23	118.3 (2)	N2—C17—C18	125.88 (19)
O2—N5—C23	118.2 (2)	N3—C17—C18	123.68 (18)
C2—C1—C6	120.0 (2)	C17—C18—H18A	109.5
C2—C1—H1	120.0	C17—C18—H18B	109.5
C6—C1—H1	120.0	H18A—C18—H18B	109.5
C3—C2—C1	120.3 (2)	C17—C18—H18C	109.5
C3—C2—H2	119.9	H18A—C18—H18C	109.5
C1—C2—H2	119.9	H18B—C18—H18C	109.5
C2—C3—C4	120.5 (2)	N4—C19—C20	118.7 (2)
C2—C3—H3	119.8	N4—C19—H19	120.7
C4—C3—H3	119.8	C20—C19—H19	120.7
C3—C4—C5	119.3 (2)	C25—C20—C21	119.3 (2)
C3—C4—H4	120.3	C25—C20—C19	122.8 (2)
C5—C4—H4	120.3	C21—C20—C19	117.8 (2)
C6—C5—C4	120.2 (2)	C22—C21—C20	120.2 (2)
C6—C5—H5	119.9	C22—C21—H21	119.9
C4—C5—H5	119.9	C20—C21—H21	119.9
C5—C6—C1	119.6 (2)	C23—C22—C21	118.4 (2)
C5—C6—C7	122.49 (19)	C23—C22—H22	120.8
C1—C6—C7	117.9 (2)	C21—C22—H22	120.8
O1—C7—C6	120.42 (19)	C22—C23—C24	123.1 (2)
O1—C7—C8	120.74 (19)	C22—C23—N5	118.9 (2)
C6—C7—C8	118.78 (18)	C24—C23—N5	118.0 (2)
C7—C8—C9	111.96 (17)	C25—C24—C23	118.0 (2)
C7—C8—H8A	109.2	C25—C24—H24	121.0
C9—C8—H8A	109.2	C23—C24—H24	121.0
C7—C8—H8B	109.2	C24—C25—C20	120.9 (2)
C9—C8—H8B	109.2	C24—C25—H25	119.5
H8A—C8—H8B	107.9	C20—C25—H25	119.5
N1—C9—C10	108.68 (16)	Cl2—C26—Cl1	104.9 (6)
N1—C9—C8	109.28 (16)	Cl2—C26—H26A	110.8
C10—C9—C8	115.60 (17)	Cl1—C26—H26A	110.8
N1—C9—H9	107.7	Cl2—C26—H26B	110.8
C10—C9—H9	107.7	Cl1—C26—H26B	110.8
C8—C9—H9	107.7	H26A—C26—H26B	108.8
C15—C10—C11	118.4 (2)	Cl4—C27—Cl3	119.9 (6)
C15—C10—C9	123.63 (18)	Cl4—C27—H27A	107.4
C11—C10—C9	118.01 (19)	Cl3—C27—H27A	107.3
C12—C11—C10	120.7 (2)	Cl4—C27—H27B	107.4
C12—C11—H11	119.6	Cl3—C27—H27B	107.4
C10—C11—H11	119.6	H27A—C27—H27B	106.9
C13—C12—C11	119.7 (2)	Cl5—C28—Cl6	128.9 (7)
C13—C12—H12	120.1	Cl5—C28—H28A	105.1
C11—C12—H12	120.1	Cl6—C28—H28A	105.1
C14—C13—C12	120.1 (2)	Cl5—C28—H28B	105.1
C14—C13—H13	120.0	Cl6—C28—H28B	105.1
C12—C13—H13	120.0	H28A—C28—H28B	105.9
C13—C14—C15	120.3 (2)	Cl7—C29—Cl8	110.7 (10)
C13—C14—H14	119.8	Cl7—C29—H29A	109.5
C15—C14—H14	119.8	Cl8—C29—H29A	109.5
C10—C15—C14	120.8 (2)	Cl7—C29—H29B	109.5
C10—C15—H15	119.6	Cl8—C29—H29B	109.5
C14—C15—H15	119.6	H29A—C29—H29B	108.1
N1—C16—N3	101.95 (16)		
			
C16—N1—N2—C17	1.0 (2)	N2—N1—C16—N3	−1.2 (2)
C9—N1—N2—C17	177.73 (17)	C9—N1—C16—N3	−177.65 (17)
C6—C1—C2—C3	−1.3 (3)	N2—N1—C16—S1	179.26 (15)
C1—C2—C3—C4	1.2 (4)	C9—N1—C16—S1	2.8 (3)
C2—C3—C4—C5	−0.3 (4)	C19—N4—N3—C17	−179.04 (18)
C3—C4—C5—C6	−0.4 (4)	C19—N4—N3—C16	1.0 (3)
C4—C5—C6—C1	0.3 (3)	N1—C16—N3—N4	−179.15 (19)
C4—C5—C6—C7	178.5 (2)	S1—C16—N3—N4	0.4 (3)
C2—C1—C6—C5	0.5 (3)	N1—C16—N3—C17	0.9 (2)
C2—C1—C6—C7	−177.69 (19)	S1—C16—N3—C17	−179.57 (16)
C5—C6—C7—O1	168.2 (2)	N1—N2—C17—N3	−0.4 (2)
C1—C6—C7—O1	−13.7 (3)	N1—N2—C17—C18	179.9 (2)
C5—C6—C7—C8	−14.6 (3)	N4—N3—C17—N2	179.70 (17)
C1—C6—C7—C8	163.60 (19)	C16—N3—C17—N2	−0.3 (2)
O1—C7—C8—C9	−10.1 (3)	N4—N3—C17—C18	−0.5 (3)
C6—C7—C8—C9	172.62 (18)	C16—N3—C17—C18	179.4 (2)
C16—N1—C9—C10	111.5 (2)	N3—N4—C19—C20	178.82 (17)
N2—N1—C9—C10	−64.8 (2)	N4—C19—C20—C25	−4.4 (3)
C16—N1—C9—C8	−121.6 (2)	N4—C19—C20—C21	177.44 (19)
N2—N1—C9—C8	62.1 (2)	C25—C20—C21—C22	1.7 (3)
C7—C8—C9—N1	161.13 (17)	C19—C20—C21—C22	179.93 (19)
C7—C8—C9—C10	−75.9 (2)	C20—C21—C22—C23	−0.5 (3)
N1—C9—C10—C15	123.7 (2)	C21—C22—C23—C24	−0.7 (3)
C8—C9—C10—C15	0.4 (3)	C21—C22—C23—N5	179.95 (19)
N1—C9—C10—C11	−55.4 (2)	O3—N5—C23—C22	−172.5 (2)
C8—C9—C10—C11	−178.63 (18)	O2—N5—C23—C22	7.6 (3)
C15—C10—C11—C12	−2.3 (3)	O3—N5—C23—C24	8.2 (3)
C9—C10—C11—C12	176.8 (2)	O2—N5—C23—C24	−171.8 (2)
C10—C11—C12—C13	1.0 (4)	C22—C23—C24—C25	0.8 (3)
C11—C12—C13—C14	0.4 (4)	N5—C23—C24—C25	−179.88 (19)
C12—C13—C14—C15	−0.6 (4)	C23—C24—C25—C20	0.4 (3)
C11—C10—C15—C14	2.1 (3)	C21—C20—C25—C24	−1.6 (3)
C9—C10—C15—C14	−176.9 (2)	C19—C20—C25—C24	−179.8 (2)
C13—C14—C15—C10	−0.8 (4)		

### Hydrogen-bond geometry (Å, °)

**Table:**

*D*—H···*A*	*D*—H	H···*A*	*D*···*A*	*D*—H···*A*
C19—H19···S1	0.95	2.43	3.199 (3)	137
C18—H18B···O1^i^	0.98	2.55	3.443 (4)	152
C22—H22···O1^ii^	0.95	2.34	3.202 (3)	151

Symmetry codes: (i) *x*−1, *y*, *z*; (ii) −*x*+1, −*y*+1, −*z*.
